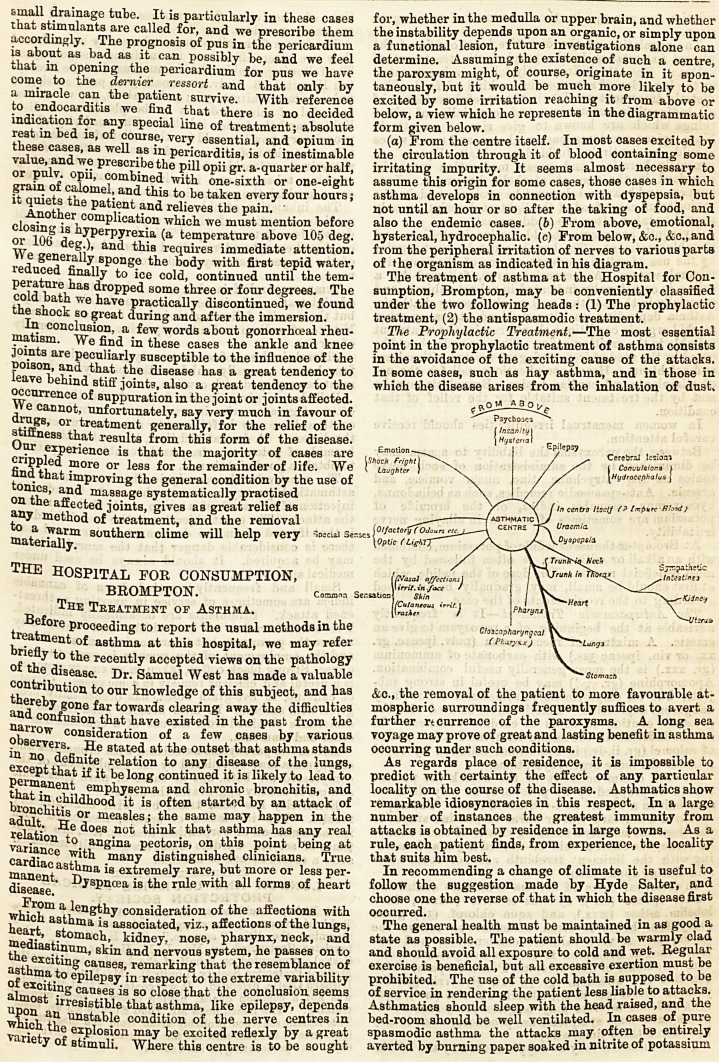# The Treatment of Asthma

**Published:** 1893-04-22

**Authors:** 


### THE HOSPITAL FOR CONSUMPTION, BROMPTON.

### The Treatment of Asthma.

Before proceeding to report the usual methods in the treatment of asthma at this hospital, we may refer briefly to the recently accepted views on the pathology of the disease. Dr. Samuel West has made a valuable contribution to our knowledge of this subject, and has thereby gone far towards clearing away the difficulties and confusion that have existed in the past from the narrow consideration of a few cases by various observers. He stated at the outset that asthma stands in no definite relation to any disease of the lungs, except that if it be long continued it is likely to lead to permanent emphysema and chronic bronchitis, and that in childhood it is often started by an attack of bronchitis or measles; the same may happen in the adult. He does not think that asthma has any real relation to angina pectoris, on this point being at variance with many distinguished clinicians. True cardiac asthma is extremely rare, but more or less permanent. Dyspnœa is the rule with all forms of heart disease.

From a lengthy consideration of the affections with which asthma is associated, viz., affections of the lungs, heart, stomach, kidney, nose, pharynx, neck, and mediastinum, skin and nervous system, he passes on to the exciting causes, remarking that the resemblance of asthma to epilepsy in respect to the extreme variability of exciting causes is so close that the conclusion seems almost irresistible that asthma, like epilepsy, depends upon an unstable condition of the nerve centres in which the explosion may be excited reflexly by a great variety of stimuli. Where this centre is to be sought for, whether in the medulla or upper brain, and whether the instability depends upon an organic, or simply upon a functional lesion, future investigations alone can determine. Assuming the existence of such a centre, the paroxysm might, of course, originate in it spontaneously, but it would be much more likely to be excited by some irritation reaching it from above or below, a view which he represents in the diagrammatic form given below.

(*a*) From the centre itself. In most cases excited by the circulation through it of blood containing some irritating impurity. It seems almost necessary to assume this origin for some cases, those cases in which asthma develops in connection with dyspepsia, but not until an hour or so after the taking of food, and also the endemic cases. (*b*) From above, emotional, hysterical, hydrocephalic. (*c*) From below, &c., &c., and from the peripheral irritation of nerves to various parts of the organism as indicated in his diagram.

The treatment of asthma at the Hospital for Consumption, Brompton, may be conveniently classified under the two following heads: (1) The prophylactic treatment, (2) the antispasmodic treatment.

*The Prophylactic Treatment.*—The most essential point in the prophylactic treatment of asthma consists in the avoidance of the exciting cause of the attacks. In some cases, such as hay asthma, and in those in which the disease arises from the inhalation of dust,

&c., the removal of the patient to more favourable atmospheric surroundings frequently suffices to avert a further recurrence of the paroxysms. A long sea voyage may prove of great and lasting benefit in asthma occurring under such conditions.

As regards place of residence, it is impossible to predict with certainty the effect of any particular locality on the course of the disease. Asthmatics show remarkable idiosyncracies in this respect. In a large number of instances the greatest immunity from attacks is obtained by residence in large towns. As a rule, each patient finds, from experience, the locality that suits him best.

In recommending a change of climate it is useful to follow the suggestion made by Hyde Salter, and choose one the reverse of that in which the disease first occurred.

The general health must be maintained in as good a state as possible. The patient should be warmly clad and should avoid all exposure to cold and wet. Regular exercise is beneficial, but all excessive exertion must be prohibited. The use of the cold bath is supposed to be of service in rendering the patient less liable to attacks. Asthmatics should sleep with the head raised, and the bed-room should be well ventilated. In cases of pure spasmodic asthma the attacks may often be entirely averted by burning paper soaked in nitrite of potassium solution (gr. xxx. ℥j.) in the bed-room before going to sleep.

The regulation of the digestive functions is of the utmost importance in the prophylactic treatment of asthma. The attack frequently follows a heavy meal, or the ingestion of some particular kind of food. The dietetic treatment consists in the careful avoidance of all indigestible substances, and especially of those things which are known to give rise to an attack. Nuts, spices, preserves, pastry, cheese, and sweet wines should be entirely forbidden. Malt liquors are generally harmful, and great moderation must be exercised in the use of alcoholic beverages. Late suppers and heavy meals must be avoided, and in the majority of cases food of the lightest description only should be taken after the midday meal.

Dyspeptic symptoms should receive immediate and suitable treatment. The bowels must be kept regular by the use of saline aperients, or one of the purgative mineral waters. A mercurial purge, followed by a saline, is occasionally indicated in robust subjects, and especially in those with a well marked gouty diathesis. Asthma depending on altered blood states or other morbid organic conditions should receive the treatment suitable to the exciting cause. Thus anæmia should be cured by the used of iron, and gout treated with salines and colchicum. Heart complications call for the use of stimulants and cardiac tonics. Bronchitis must be met by the treatment suitable for the relief of that condition.

In women menstrual irregularities should receive careful attention.

Between the paroxysms the liability to spasm may be diminished by the administration of such nerve tonics as Liq. strychniæ, tinc. nucis vomicæ, and arsenic. Anti-spasmodic remedies, such as belladonna, hyoscyamus, cannabis indica, or the bromide of potassium are sometimes of service in allaying nerve irritability.

At Brompton the use of iodide of potassium, combined with an alkali or with iron, is often followed by the most beneficial results. The dose of the iodide, which at first should be small, may gradually be increased according to the requirements of the case.

*The Antispasmodic Treatment.*—It is frequently advisable at the beginning of a paroxysm to give an emetic. A mixture of ipecacuanha (pulv. ipecac. gr. xx. or vin. ipecac ℥ss.) with carbonate of ammonium (gr. xxx.) is the most generally useful combination. Apomorphine (gr. $\frac{1}{10}$-$\frac{1}{6}$) may be useful in strong subjects, but its employment is sometimes followed by dangerous depression. Emetics must be used with the greatest caution in cases of cardiac weakness. In robust individuals the administration of a few grains of calomel (gr. ii.-iv.) is sometimes followed by rapid relief of the more urgent symptoms.

At the commencement of the attack the patient should be propped up by pillows, or he may sit in an armchair in a warm, well-ventilated room. The application of a mustard leaf or warm turpentine stupes to the chest has usually a soothing effect. If these cannot be borne, the chest should be rubbed night and morning with the liniment terebinth acet, or some other stimulating application.

In catarrhal cases of asthma a mixture containing vinum ipecac (mx), spiritus ætheris (mxxx.), tinct. lobeliæ æther (mxx.), and aqua chlorof. (℥j.) given every four hours is often sufficient to relieve the paroxysm. A very valuable combination, and one much used at Brompton is the mistura potassii iodidi cum stramonio of the Hospital Pharmacopœia, which contains ext. stramon. (gr. $\frac{1}{4}$), ext. glycyrrh. (gr. ii.), potassii iodidi (gr. iii-x.), ætheris chlorici (mv.), aqua (℥j.), and this mixture may be given every four or six hours until toxic symptoms are produced, when it may be ordered three times a day.

These measures may be supplemented by the inhalation of the fumes from various sedative and anti-spasmodic powders.

The fumes from nitre paper alone may be used, and they are often efficacious in subduing the paroxysm. The following combination is much used at the Brompton Hospital: ℞. Pulv. stramonii, ʒiv.; pulv. anisi, ʒii.; pulv. potassii nitrat, ʒii.; pulv. tabaci, gr. v. A teaspoonful of this powder is to be placed on a dish, lighted, and the fumes inhaled. The majority of the proprietary powders sold for the relief of asthma contain some plant of the order Atropaceæ with nitrate of potassium. Considerable relief may be obtained by smoking tobacco, and inhaling the fumes, in those cases in which the patient is unaccustomed to its use.

The inhalation of chloroform is followed by an immediate cessation of the paroxysm, but the benefit derived is only temporary. This drug should never be employed except under medical supervision, on account of the dangers that may result from its indiscriminate use.

Temporary relief is also, as a rule, obtained by the administration of nitrite of amyl or iodide of ethyl, but the results following their use are not constant. Nitrite of sodium and nitro-glycerine are occasionally of service.

Chloral, in doses of ten to twenty grains, given every four hours, generally subdues the paroxysm, but it must be employed with caution.

Belladonna, given in the form of the tincture, is often of great service in averting an attack, and its continued use may be followed by a permanent cure of the disease. A dose at bed time, in combination with bromide of potassium, frequently enables the patient to obtain a good night's rest.

The most valuable remedy in the treatment of the asthmatical paroxysm is morphia. A hypodermic injection of one-sixth of a grain rarely fails to relieve the attack. The great objection to the use of this drug is that if used often the dose must be increased, and there is considerable danger that the morphia habit may be acquired. It should always be used under medical supervision.

Small and frequently-repeated doses of cannabis indica are sometimes of service in averting a threatened attack. Stimulants are occasionally indicated. The administration of hot whisky and water may relieve the paroxysm, but tolerance is soon established, and the employment of the remedy is open to grave abuse. Strong black coffee, taken on an empty stomach, is a useful and efficient remedy in some cases. Citrate of caffeine, in five-grain doses, has occasionally been given with success. In mild attacks good results are sometimes obtained from the use of a combination of ether and ammonia.

Asthmatic paroxysms associated with bronchitis and emphysema may be relieved by the employment of the compressed air bath, but in cases of pure spasmodic asthma it usually aggravates the attack.

Electricity has been tried, but with contradictory results. The inhalation of sprays, medicated with ipecacuanha, oil of turpentine, arsenic, &c., are sometimes of service. Cardiac tonics are generally indicated after a severe attack of asthma.

### LONDON AND COUNTIES MEDICAL PROTECTION SOCIETY.

At Bishop Auckland on Thursday. April 13th, William Todd, of Evenwood, was charged with wilfully and falsely assuming the title "surgeon." Mr. H. Gawan-Taylor, barrister (instructed by Mr. J. H. Holmes, of Barnard Castle), prosecuted on behalf of the London and Counties Medical Protection Society (Limited). Mr. Barmingham, of Barnard Castle, defended. It was contended that Mr. Todd, was not a common quack, having received some medical training. The Bench fined Mr. Todd £1, and £2 2s. costs. This lenient sentence probably arose from the magistrates' ignorance of the fact that the fine is intended to cover the expense of prosecuting in such cases.

## Figures and Tables

**Figure f1:**